# The role of KCC2 and NKCC1 in spinal cord injury: From physiology to pathology

**DOI:** 10.3389/fphys.2022.1045520

**Published:** 2022-12-15

**Authors:** Zuliyaer Talifu, Yunzhu Pan, Han Gong, Xin Xu, Chunjia Zhang, Degang Yang, Feng Gao, Yan Yu, Liangjie Du, Jianjun Li

**Affiliations:** ^1^ School of Rehabilitation, Capital Medical University, Beijing, China; ^2^ Department of Spinal and Neural Functional Reconstruction, China Rehabilitation Research Center, Beijing, China; ^3^ Chinese Institute of Rehabilitation Science, Beijing, China; ^4^ Center of Neural Injury and Repair, Beijing Institute for Brain Disorders, Beijing, China; ^5^ Beijing Key Laboratory of Neural Injury and Rehabilitation, Beijing, China; ^6^ School of Rehabilitation Sciences and Engineering, University of Health and Rehabilitation Sciences, Qingdao, China

**Keywords:** spinal cord injury, CCCS, KCC2, NKCC1, neuropathic pain, spasticity, motor function reconstruction

## Abstract

The balance of ion concentrations inside and outside the cell is an essential homeostatic mechanism in neurons and serves as the basis for a variety of physiological activities. In the central nervous system, NKCC1 and KCC2, members of the SLC12 cation-chloride co-transporter (CCC) family, participate in physiological and pathophysiological processes by regulating intracellular and extracellular chloride ion concentrations, which can further regulate the GABAergic system. Over recent years, studies have shown that NKCC1 and KCC2 are essential for the maintenance of Cl^−^ homeostasis in neural cells. NKCC1 transports Cl^−^ into cells while KCC2 transports Cl^−^ out of cells, thereby regulating chloride balance and neuronal excitability. An imbalance of NKCC1 and KCC2 after spinal cord injury will disrupt CI^−^ homeostasis, resulting in the transformation of GABA neurons from an inhibitory state into an excitatory state, which subsequently alters the spinal cord neural network and leads to conditions such as spasticity and neuropathic pain, among others. Meanwhile, studies have shown that KCC2 is also an essential target for motor function reconstruction after spinal cord injury. This review mainly introduces the physiological structure and function of NKCC1 and KCC2 and discusses their pathophysiological roles after spinal cord injury.

## Introduction

Spinal cord injury (SCI) is a commonly occurring disabling disease that can lead to different degrees of impairment of motor, sensory, and autonomic functions in the segment below the site of injury (McDonald and Sadowsky, 2002). Nearly one million new spinal cord injuries occur annually worldwide, imposing a heavy burden on the families of patients and society as a whole (Cieza et al., 2021; GBD 2016 Neurology Collaborators, 2019a, GBD 2016 Traumatic Brain Injury and Spinal Cord Injury, 2019b). After SCI, in addition to damage to the motor and sensory pathways, injury to other pathways and the adaptive changes in the damaged lower segment result in profound changes in the microenvironment of the spinal cord (O'Shea et al., 2017). Complications such as spasticity and neuropathy can also seriously affect the mood, sleep, quality of life, cognitive function, recreational activities, and even the employment of patients, and can also dramatically affect the recovery process (O'Shea et al., 2017; Finnerup, 2017). Currently, the treatment of direct dysfunction and complications caused by SCI is relatively limited, and the individual differences are significant (Ahuja et al., 2017). A lack of a detailed understanding of the physiological mechanism of spinal cord regulation and the pathophysiological process occurring after SCI injury greatly contributes to the scarcity of available treatment options (McDonald and Sadowsky, 2002; Ahuja et al., 2017).

Over recent years, potassium-chloride co-transporter 2 (KCC2) and sodium-potassium-chloride co-transporter 1 (NKCC1) have been increasingly recognized as playing important roles after SCI (Kaila et al., 2014; Tang, 2020). These two co-transporters proteins are known to exert critical regulatory effects in the recovery from spasticity and neuropathic pain as well as the restoration of motor function after SCI (Kaila et al., 2014; Chen et al., 2018; Côme et al., 2020). This review mainly introduces the physiological functions of KCC2 and NKCC1 and the research progress on their roles in spasticity, neuropathic pain, and motor function recovery following injury to the spinal cord.

## Overview of KCC2/NKCC1

Cation-chloride co-transporters (CCC) mediate the coupled movement of Cl^−^, K^+^, and/or Na^+^ across the plasma membrane, which underlies the regulation of intracellular Cl^−^ concentrations and plays an important role in neuronal excitability, transepithelial salt and water movement, and the regulation of cell volume (Xie et al., 2020; Zhang et al., 2021). There are two main subfamilies of CCCs, namely, Na^+^ (K^+^)-coupled transporters [NKCC1, NKCC2 and NCC (SLC12A1–3)] and K^+^-coupled transporters [KCC1–4 (SLC12A4–7)] (Russell, 2000; Arroyo et al., 2013).

### The structure and expression of KCC2

KCC2 is the major potassium concentration gradient-driven K^+^-Cl^−^ efflux co-transporter, functioning to reduce the intracellular Cl^−^ concentration, which is encoded by the solute carrier family 12 member 5 (*SLC12A5*) gene and consists of 12 transmembrane domains (TMDs) and intracellular N and C termini (Payne et al., 1996). In addition, KCC2 contains an extracellular domain (ECD), formed by a large loop between transmembrane helix 5 (TM5) and TM6, and a C-terminal domain (CTD) immediately following TM12 (Payne, 2012; Xie et al., 2020). The CTD regulates the expression, transport, and activity of KCC2 *via* phosphorylation and dephosphorylation (Lee et al., 2010). KCC2 has two isoforms—KCC2a and KCC2b—that differ in their N termini and are regulated by different promoters (Agez et al., 2017). Of the two isoforms, the expression of KCC2a is relatively low, KCC2b plays the major role in the mature cerebral cortex, hippocampus, and cerebellum (Markkanen et al., 2014). KCC2b expression is significantly up-regulated in the cerebral cortex and cerebellum of rats within 1 week of birth, and can account for 90% of the total KCC2 protein content in the cortex in adulthood (Uvarov et al., 2009). However, in the spinal cord, the two isoforms are expressed at similar levels, and have similar but not completely overlapping distribution patterns in the anterior and posterior spinal cords (Uvarov et al., 2009; Markkanen et al., 2014). When KCC2 was knocked out, mice developed generalized epilepsy and died shortly after birth from severe motor and respiratory deficits (Hübner et al., 2001). However, when the KCC2b isoform was selectively knocked out, the mice survived for 3 weeks after birth (Woo et al., 2002). The overexpression of KCC2a resulted in a significant negative shift in γ-aminobutyric acid (GABA) reversal potential, like that seen with KCC2b overexpression (Markkanen et al., 2017). This suggested that the physiological function of the KCC2a subtype is similar to that of KCC2b and can compensate for its physiological function, at least to some extent.

### The structure and expression of NKCC1

NKCCs belong to the CCC family and mediate the transport of Na^+^, K^+^, and Cl^−^ ions into cells. NKCC1 and NKCC2 are the only two currently known NKCC family members (Haas and Forbush, 2000). In humans, NKCC1 is encoded by the *SLC12A2* gene located on chromosome 5q23 (Markadieu and Delpire, 2014). Like KCC2, NKCC1 also has 12 α-helical transmembrane domains that are flanked by long non-hydrophobic domains at the N and C termini. NKCC1 contains a total of 1,212 amino acid residues and a relative molecular mass of 131.4 kDa (Gamba et al., 1994; Markadieu and Delpire, 2014). The NKCC1 protein is widely expressed in central and peripheral neurons, especially in the cerebral cortex, striatum, hippocampal pyramidal cells, glial cells, and spinal dorsal root ganglia (Pond et al., 2006; Pitcher et al., 2007; Krystal et al., 2012). In general, NKCC1 expression is much higher in glial cells than in neurons (Virtanen et al., 2020). In addition, NKCC1 is widely distributed in cells of the cardiovascular system, the outer plexiform layer of the distal retina, and the skeletal muscle system (Jaggi et al., 2015). NKCC2 isoforms are only found in renal medullary regions, loops, and periglomerular organs (Payne and Forbush, 1995). In the early stages of mammalian development, the expression of NKCC1 in the central nervous system is higher than that of KCC2 (Medina et al., 2014). As development progresses, the expression of NKCC1 gradually decreases, whereas that of KCC2 gradually increases, resulting in a change in GABA activity from depolarizing to hyperpolarizing (Ben-Ari, 2002; Di Cristo et al., 2018).

### The physiological function of KCC2 and NKCC1

KCC2 is the primary Cl^−^ export mechanism in mature mammalian neurons, driving intracellular Cl^−^ concentrations below their electrochemical equilibrium potential, thereby enhancing GABA_A_ hyperpolarization and postsynaptic inhibition levels (Coull et al., 2003; Kahle et al., 2014). In contrast, NKCC1, expressed in afferent neurons, drives the intracellular concentration of Cl^−^ above its equilibrium potential, thereby promoting primary afferent depolarization and presynaptic inhibition, which is critical for gating sensory information from the peripheral to the central nervous system (Kahle et al., 2014; Kaila et al., 2014). The neurotransmitter GABA mainly regulates neuronal excitability through GABA type A receptors (GABA_A_Rs), which are ligand-gated Cl^−^ channels (Kahle et al., 2008). The effect of GABA_A_Rs on cell function depends on the relative concentration of intracellular Cl^−^, and the membrane potential is determined by intracellular and extracellular Cl^−^ concentrations and remains relatively constant, when GABA_A_R channels open, the membrane potential is pulled toward the Cl^−^ equilibrium potential (Chamma et al., 2012). In settings of low KCC2 and high NKCC1 activity, such as in early development or in specific neuropathic pain states, the Cl^−^ influx mechanism overrides the KCC2-mediated Cl^−^ efflux, resulting in high intracellular Cl^−^ concentrations, and GABA_A_R activation results in depolarization (Kahle et al., 2014; Tillman and Zhang, 2019). When KCC2 activity is high and NKCC1 activity is low, such as in healthy, mature neurons, KCC2-mediated efflux maintains a low intracellular Cl^−^ concentration, enabling GABA_A_R activation and leading to neuronal hyperpolarization (Tillman and Zhang, 2019).

GABA is the primary inhibitory neurotransmitter in the central nervous system of mature mammals, and its primary role is the reduction of the excitability of neurons, and also involved in regulating muscle tone through the modulation of nerve impulses (Watanabe et al., 2002). These physiological roles of GABA are exerted in an intracellular chloride concentration-depend manner (Kaila et al., 2014; Chamma et al., 2012). Meanwhile, it has been reported that GABA_A_R activity can also affect the function of KCC2 (Heubl et al., 2017). GABA_A_R activation can reduce the diffusion coefficient of KCC2, thereby increasing its membrane concentration and stability, while GABA_A_R blockers can increase the fluidity of KCC2 as well as reduce its density, stability, and activity on the surface of the cell membrane (Heubl et al., 2017). Moreover, GABA_A_R blockers can also induce the phosphorylation of NKCC1 through the STE20/SPS1-related proline/alanine-rich kinase (SPAK) and oxidative stress-responsive kinase 1 (OSR1) pathway (Heubl et al., 2017). KCC2, NKCC1, and the GABA system affect the activity of the nervous system under a variety of physiological conditions, with intracellular Cl^−^ concentrations serving as the intermediate link.

In addition to their function as ion transporters, KCC2 and NKCC1 also have cell-autonomous regulatory functions in the central nervous system, including the regulation of GABA_A_R and glycine receptor-mediated reversal potentials and anion currents (Blaesse et al., 2009), as well as physiological functions such as the regulation of cell volume, ion homeostasis, and growth (Olde Engberink et al., 2018; Demian et al., 2019). Studies have shown that the effects of NKCC1 and KCC2 on the regulation of cell osmotic pressure and cell volume are mediated through their ion transport functions (Chew et al., 2019). Meanwhile, KCC2 and NKCC1 also act as synchronization factors during the development of glutamatergic and GABAergic synapses in cortical neurons and their networks (Di Cristo et al., 2018). In addition, the importance of these two co-transporters in GABAergic signaling renders them valuable targets for nervous system drugs, such as those aimed at treating neuropathic pain, spasm, epilepsy, and motor dysfunction, among other conditions.

### KCC2/NKCC1 and neurodevelopment

NKCC1 and KCC2 jointly regulate Cl^−^ homeostasis in neurons and thus affect the function of the GABAergic system (Côme et al., 2020). In the early stages of development, because the expression level of KCC2 is relatively low and that of NKCC1 relatively high, intracellular Cl^−^ concentrations remain at a relatively high level (Medina et al., 2014). Under this condition, the GABA_A_R can play an excitatory role by extruding Cl^−^ from the cell (Medina et al., 2014). As the nervous system develops and matures, the concomitant increase in the expression of KCC2 results in a gradual reduction in the intracellular Cl^−^ concentration, and the function of the GABA_A_R changes accordingly, which promotes Cl^−^ influx, thereby exerting an inhibitory role (Côme et al., 2019). It has been demonstrated that if the spinal cord is transected during development, the expression of KCC2 is not up-regulated, and the transition of GABA function does not occur (Jean-Xavier et al., 2006). This suggests that the transition of GABA function from depolarizing to hyperpolarizing is related to the maturation of relevant spinal cord pathways. Notably, although it is generally assumed that NKCC1 is downregulated and KCC2 up-regulated during neuronal development, some existing data do not support this generalization (Blaesse et al., 2009). For instance, it cannot be excluded that a relatively small pool of NKCC1 protein in the plasma membrane of immature neurons may be sufficient to exert a dominant effect on the GABA system (Löscher et al., 2013). Additionally, the marked upregulation of KCC2 during development may be accompanied by a substantial increase in ionic conductivity and neuronal growth, which can mask the effect of NKCC1, even if the expression of the latter remains unchanged (Loscher et al., 2013). The transition of GABA function is related to the phosphorylation of serine 940 (Ser-940, S940), an important site for KCC2 activation (Leonzino et al., 2016). In parallel to the up-regulating signaling pathways, there are two threonine residues, Thr-906 and Thr-1007, on the intracellular C-terminal domain of KCC2 that strongly decrease KCC2 activity when phosphorylated (Schulte et al., 2018). Specifically blocking the function of KCC2 can induce a polarity change in GABAergic responses from inhibitory to excitatory. During development, With-No-Lysine kinase 1 (WNK1) -dependent regulation of KCC2 and NKCC1 phosphorylation levels is also an essential basis for the functional transformation of GABA (Gagnon and Delpire, 2010; Lee et al., 2011). WNK is mainly affected by intracellular Cl^−^ concentration and may play a regulatory role on KCC2 and NKCC1 through the oxidative stress-responsive gene 1/Ste20-related proline-alanine-rich kinase (OSR1/SPAK) pathway (Friedel et al., 2015). Combined, these findings suggest that the KCC2 and NKCC1 functions may play a critical role in the change of GABA function during development (Olde Engberink et al., 2018).

NMDA receptor (N-methyl-D-aspartic acid receptor) is a subtype of ionic glutamate receptor; its role in neuroplasticity and excitotoxicity has received increasing attention (Vyklicky et al., 2014). *In vitro* and *in vivo* studies revealed that NMDA receptor activity-mediated protein phosphatase 1 (PP1) can inhibit the function of NKCC1 and upstream factors by regulating their dephosphorylation (Gagnon and Delpire, 2010), and the dynamic balance between PP1-dependent dephosphorylation of SER940 at the key site of KCC2 and regulation of protein kinase C (PKC) is an important basis for KCC2 to ensure physiological functions (Lee et al., 2011).

Growth factors play an indispensable role in the construction of neural networks during neural development and regeneration. For instance, brain-derived neurotrophic factor (BDNF) is a key regulator of axon growth, synapse formation, intersynaptic signal transmission, and synaptic plasticity (Wardle and Poo, 2003; Garraway and Huie, 2016; Lee-Hotta et al., 2019). The activation of BDNF affects the levels of intracellular Cl^−^, which, in turn, affects the function of GABA receptors in various physiological processes in the central nervous system (Zhou et al., 2021). The downstream signaling of Tyrosine Kinase receptor B (TrkB), a major receptor for BDNF, mainly involves 1) Mitogen-activated protein kinases/extracellular regulated kinase (MAPK/ERK) (Uvarov et al., 2006; Revest et al., 2014); 2) Extracellular regulated kinase/Early Growth Response 4 (ERK/Egr4) (Ludwig et al., 2011a; Ludwig et al., 2011b); and 3) Phospholipase C gamma (PLCγ)/FRS-2 (Rivera et al., 2004) (as shown in Figure 2). Among these three downstream signaling pathways, Egr4 is a transcription factor on the promoter of KCC2, which may lead to the upregulation of KCC2 mRNA after its translation and expression (Ludwig et al., 2011a; Ludwig et al., 2011b). Activation of PLCγ seems to be the key to the regulation of KCC2 by the BDNF-TrkB pathway. When PLCγ increases, BDNF inhibits KCC2 expression, while the opposite effect occurs when PLCγ activation is depressed. After SCI, the PLCγ cascade of the BDNF-TrkB pathway is enhanced, thereby repressing KCC2 mRNA transcription (Rivera et al., 2004; Tashiro et al., 2015).

BDNF is a crucial regulator of KCC2.In mature neurons, the effect of BDNF on KCC2 is inhibited. However, after SCI, the effect of BDNF on KCC2 turns into upregulated. It is may be caused due to a reversal way that BDNF affects KCC2 (Boulenguez et al., 2010). Previous studies have shown that exercise therapy such as treadmill training may endogenously increase the activation of the BDNF-TrkB pathway on injured neurons. Then, activating downstream signaling pathways and regulating KCC2/NKCC1 expression, and effectively improve the spasticity and pain after SCI (Tashiro et al., 2015). And this effect can be inhibited by the BDNF blocker TrkB-IgG, suggesting that activation of BDNF is critical to relief from spasticity and pain symptom after SCI (Bilchak et al., 2021).

Thus, BDNF may play opposing roles in the intact, mature nervous system and the injured, immature nervous system (Huang et al., 2017). There is currently no strong evidence indicating that BDNF directly affects NKCC1 expression. How to further explore this dual role of BDNF and apply it to rehabilitation after SCI requires further investigation.

**FIGURE 1:**
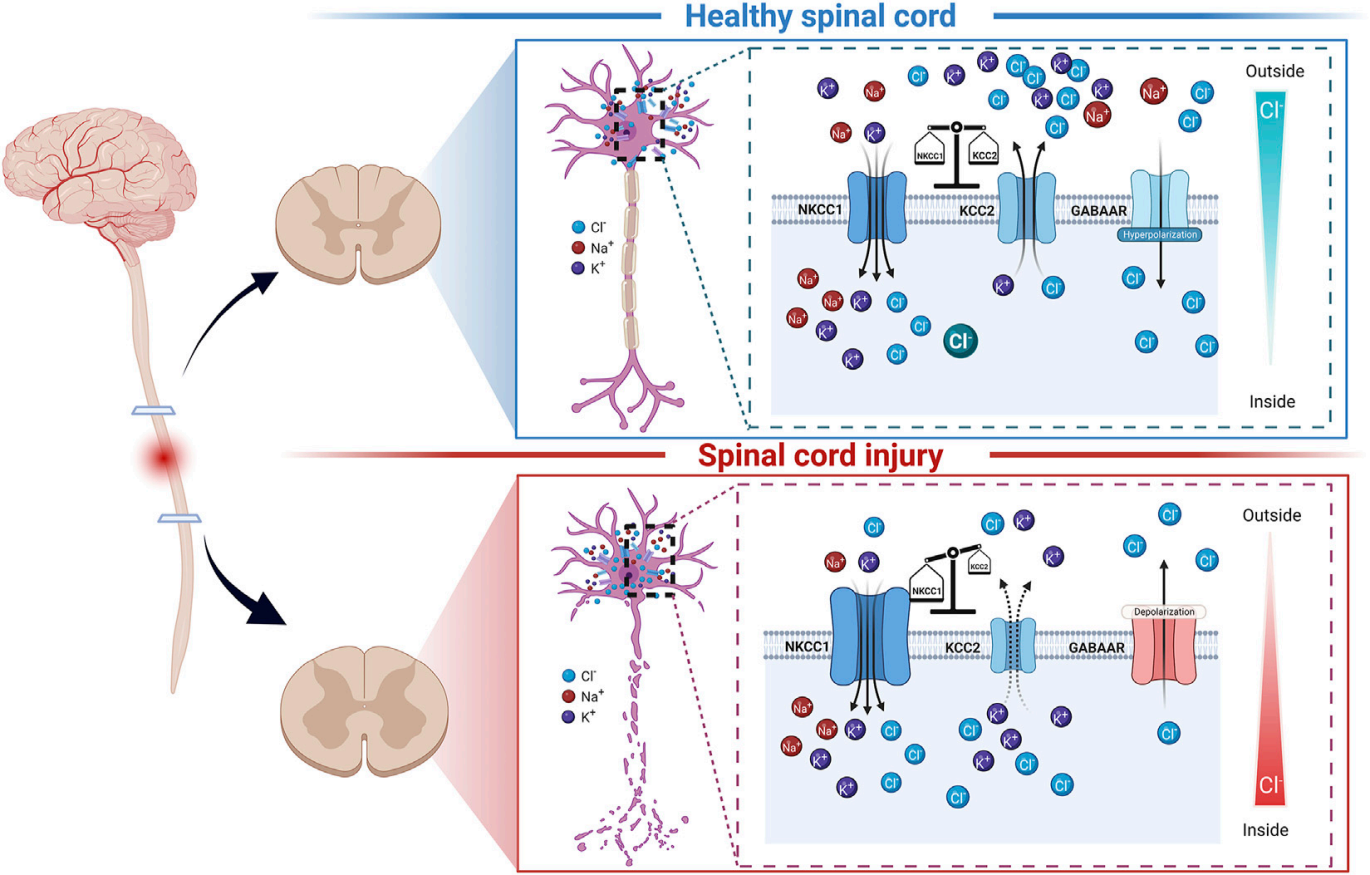
Schematic diagram of NKCC1, KCC2, GABA_A_Rs, and chloride ion homeostasis in spinal cord neurons under normal conditions and after spinal cord injury. In the mature, healthy spinal cord, the expression of NKCC1 and KCC2 is relatively balanced (top panel). Under this condition, the intracellular chloride ion level is low. GABA_A_Rs can play an inhibitory role by transporting chloride ions into cells. When spinal cord injury occurs (bottom panel), the expression of NKCC1 and KCC2 in the segment below the injury is dysregulated under the action of various factors, leading to high intracellular chloride ion levels. In this case, the GABA_A_Rs can play an inhibitory role by excluding chloride ions from cells.

**FIGURE 2:**
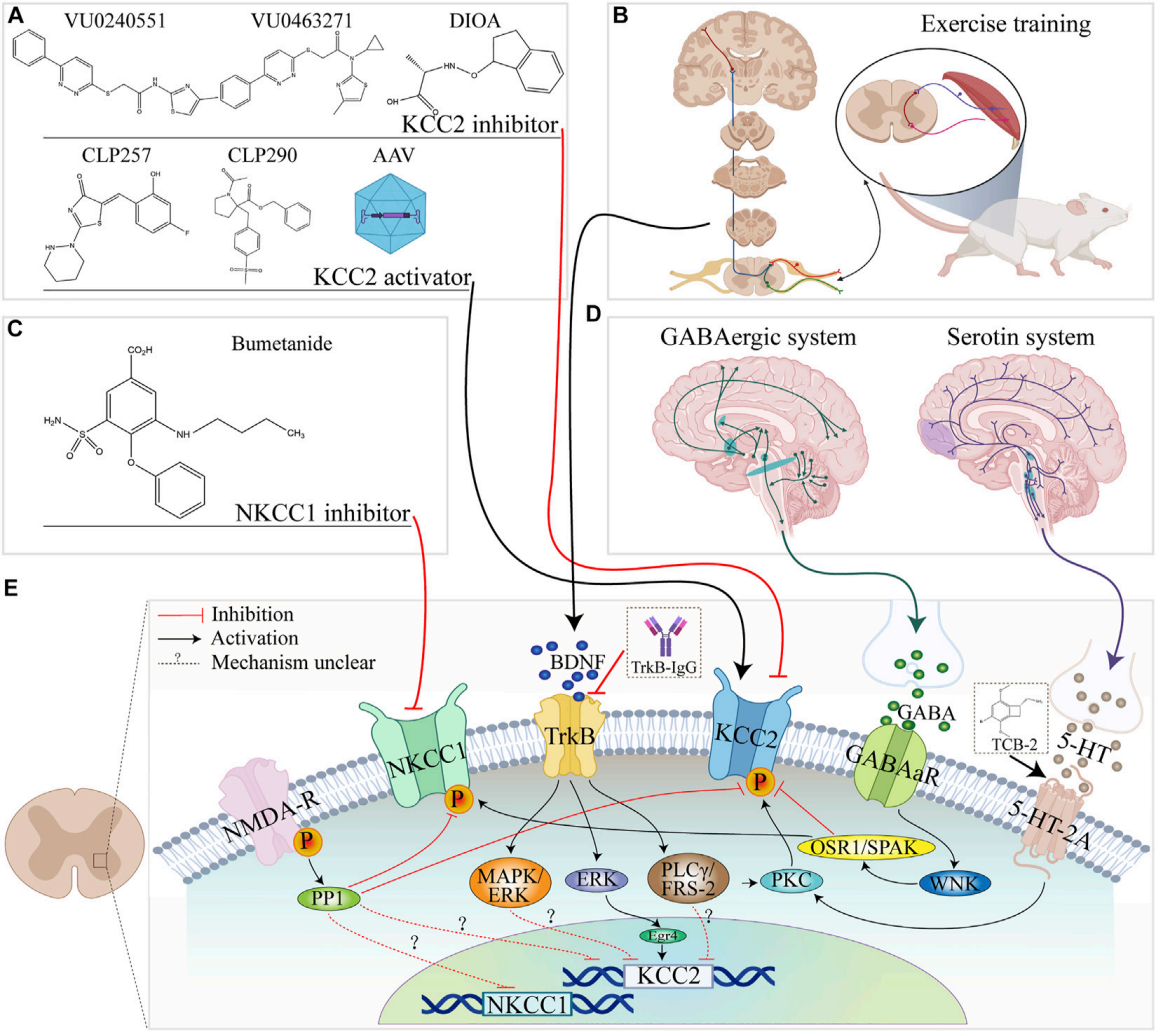
Summary of NKCC1 and KCC2 regulatory pathways in the spinal cord and related pathways. **(A)** The molecular formulae of commonly used KCC2 activators and inhibitors. **(B)** Exercise training. **(C)** Molecular formula of the NKCC1 inhibitor bumetanide. **(D)** Brain origin of 5-HT and GABAergic systems. **(E)** Schematic diagram of the relationship between KCC2/NKCC1 and upstream factors, channels, and pathways in spinal cord.

In conclusion, as shown in Figures 1, 2, the regulatory effect of BDNF on the KCC2 and GABAergic systems differs according to the developmental stage and physiological condition (Tashiro et al., 2015; Heubl et al., 2017; Ma et al., 2021). In the mature nervous system, the expression level of KCC2 is high, and GABA can reduce the excitability of the system (Heubl et al., 2017). Under this condition, exogenous BDNF application will downregulate the expression of KCC2 (Huang et al., 2017). However, when the nervous system is immature or SCI occurs, the expression level of KCC2 is low, and GABA plays an excitatory role. Here, BDNF application will increase the expression level of KCC2 and restore the inhibitory effect of GABA.

## KCC2/NKCC1 and spinal cord injury

After SCI, the segment below the site of injury presents a state similar to that seen in the early stages of development (Huang et al., 2017). KCC2 and NKCC1 are reportedly involved in the pathophysiology of SCI, including the appearance of spasticity and neuropathic pain, through a variety of pathways (Boulenguez et al., 2010; Hasbargen et al., 2010). Damage to the descending serotonin (5-HT) fibers after SCI is closely related to the transformation of GABAergic interneuron function and KCC2/NKCC1 may be the common link between the two regulatory imbalances after SCI (Huang and Grau, 2018; Sánchez-Brualla et al., 2018; Tang, 2020). BDNF can also directly participate in this process upstream of KCC2/NKCC1 (Garraway and Huie, 2016; Ma et al., 2021). The treatment of SCI by directly or indirectly regulating KCC2/NKCC1 activity represents a potential strategy for the treatment of SCI, as summarized in Table1 and Figure 2.

**TABLE 1:** Summary of animal experiments related to the regulation of KCC2 and NKCC1 after spinal cord injury.

Ref	Experimental animals	Experimental models	Treatment		Main outcome
Boulenguez et al. (2010)	• Adult Wistar rats, female • adult heterozygous mice (Slc12a5^+/−^, males)	• Spinal thoracic transection and contusion injury at T9	• DIOA	• i.t. 20 µg	• The expression of KCC2 in lumbar motoneurons is reduced after SCI
			• BDNF	• i.t. 10 µg	• Blocking KCC2 decreases the excitability of the network
Bilchak et al. (2021)	• Adult female Sprague Dawley rats	• Spinal cord transection at T12	• CLP257	• injecting on the lumbar enlargement of the spinal cord	• Increasing KCC2 activity has a beneficial effect on spasticity after SCI
			• VU0240551	• injecting on the lumbar enlargement of the spinal cord	• Enhancing KCC2 can decrease hyperreflexia after chronic SCI
			• bike-trained	• Beginning 4–5 days post-injury, 20 min, 5 days/week	• CLP257 increases the expression of KCC2 in lumbar motoneurons
Côté et al. (2014)	• Sprague Dawey Rat	• spinal cord transection at T12	• Exercise	• Beginning on days 4–5,60 min/day,5 days/week, until 14/28 days after SCI	• Exercise restores chloride homeostasis after SCI
			• DIOA	• exposed lumbar enlargement,30 mM	• A decrease in NKCC1 levels and an increase in KCC2 levels lead to reflex recovery after SCI
			• Bumetanide	• i.p. 30 mg/kg	• The GABAergic system is modulated by KCC2 and NKCC1
Bos et al. (2013)	Newborn rats	• Vitro: sacral segments up to T8–T9 and L3–L5 dorsal and ventral roots	• DOI	• 10 μM	• Active 5HT2R restores chloride homeostasis after SCI *via* Ca^2+^ dependent PKC
			• Ketanserin	• 10 μM	• Active 5HT2AR reduces spasticity after SCI
	Hb9::eGFP transgenic mice				• TCB-2 modulates chloride homeostasis and increases postsynaptic inhibition
			• TCB-2	• 0.1 μM, 10 μM	
			• VU0240551	• 25 μM	• 5HT2AR and 5HT2B/2 C R have opposing effects on the KCC2 function
Chen et al. (2018)	• Adult female WT mice at the age of 8 weeks	• T7 and T10 double lateral hemisection • T8 full transection	• Quipazine, 8-OH-DPAT,	• intraperitoneal injection	• KCC2 expression in inhibitory neurons leads to functional recovery
			• Baclofen,		• Restoring inhibition in the spinal cord after SCI leads to functional recovery
			• CP101606,		
			• CLP290,		• Reducing the excitability of spinal cord inhibitory interneurons enhances the responsiveness of injured spinal cords to descending inputs and promotes functional recovery after SCI
			• L838,417,		
			• Bumetanide saline		
	• Adult female WT mice at the age of 8 weeks • Vgat-Cre mice • Vglut2-Cre mice • ChAT-Cre mice	• T7 and T10 double lateral hemisection • T8 full transection	• AAV2/PHP.B-Syn-HA-KCC2	• tail vein injection, 200 ul • Lumbar intraspinal injection (L2-4), 0.5-1x1013 copies/ml	
			• AAV2/9-Syn-HA-KCC2		• A KCC2 agonist restores the stepping ability paralyzed mice with SCI
			• AAV2/9-Syn-FLEX-HA-KCC2		
			• AAV2/9-Syn-FLEX-hM4Di-mCherry • AAV2/9-Syn-FLEX-hM3Dq-mCherry		
Sánchez-Brualla et al. (2018)	• Adult female Wistar rats (230 ± 30 g)	• SCI model: a left hemisection at the thoracic T8 • SNI model: on the right hindlimb	• TCB-2• DIOA	• teatment1:i.p. injection of TCB-2 (0.3 mg/kg) on post-operative day 21 and 14	• TCB-2 actives 5HT2AR by increasing the membrane expression of KCC2 in the dorsal horn, thereby alleviating neuropathic pain after spinal cord hemisection
				• treatment2:intrathecal DIOA injection 20 min before TCB-2 treatment	
				• treatment3:TCB-2 (0.3 mg/kg), i.p. daily for 7 days	
Tashiro et al. (2015)	• Female Sprague-Dawley rats (8–9 weeks)	• T10 laminectomy, 200 Kd contusive injury	• TrkB-IgG	• i.t. 3 µg/day for 2 weeks	• Treadmill training can alleviate spasticity and allodynia by increasing the expression of BDNF in the lumbar enlargement
			• treadmill training		• Increasing endogenous BDNF levels by treadmill training increases the expression of KCC2
			• CLP257	• 7 days/week for 2 consecutive weeks, speed is 2.5–3.5 cm/s	• Restoring the level of KCC2 contributes to the amelioration of spasticity and allodynia
			• VU0240551		
Liabeuf et al. (2017)	• Adult female Wistar rats	• spinal cord transection at T8	• PCPZ	• i.v. 10 μg/kg	• Phenothiazine piperazine derivatives upregulate KCC2 function
			• baclofen	• i.v. 2 mg/kg	• PCPZ enhances inhibition and decreases spasticity after SCI by modulating the expression of KCC2
			• DIOA	• i.t. 40 µg	• PCPZ plays the same role as baclofen in reducing spasticity
Beverungen et al. (2020)	• Adult female Sprague-Dawley rats	• Completely thoracic transection injury at T12	• VU0240551	• 50 mM	• Decreasing spinal hyperexcitability and spasticity requires the activity of KCC2
			• TrkB-IgG		• Decreasing the activity of BDNF alleviates spinal hyperexcitability and spasticity
			• bicycling session	• Beginning on day 5, 60 min/day	• BDNF signaling is required for increasing the expression of KCC2 after SCI
					• NKCC1 and KCC2 protein levels are reciprocally regulated
Liao et al. (2022)	• Adult male Sprague Dawley (SD) rats	• spinal cord transection at T10	•LIFU	• 4 weeks	• LIFU treatment decreases spasticity
					• LIFU activates spinal neurocircuits
					• LIFU treatment upregulates the expression of KCC2 after SCI
Huang et al. (2017)	• Male Sprague-Dawley rats	• Spinally transected and cannulized at T2	• Bicuculline	• i.t. 0.3 μg	• Increasing the activation of the GABAAR maintains the capsaicin-induced EMR and modulates central sensitization
			• LSP	• i.t. 100 μg	• GABAergic neurons maintain central sensitization after SCI
			• Gabazine	• i.t. 0.0 μg/0.001 μg/0.01 μg	• Spinal transection reduces the expression of KCC2
			• DIOA	• i.t. 20 μg	• GABA function can be altered by reducing the concentration of membrane-bound KCC2
			• bumetanide	• i.t. 1 mM	
Ma et al. (2021)	• Adult male Kunming mice	• SNI by transected a 2–3 mm portion of the tibial and common peroneal nerves were	• BDNF	• i.t., 0.5 μg	• KCC2 expression in the spinal cord dorsal horn can be decreased by BDNF *via* inducing KCC2 ubiquitination
					• The knockdown of Cbl-b expression decreases KCC2 ubiquitination levels and attenuates BDNF-induced pain hypersensitivity
Plantier et al. (2019)	• Neonatal rats within the first 12 h after birth	• Spinal cord transection at the T8-T9	• MDL28170	• i.p., 60 mg/kg or 120 mg/kg	• Calpain regulates INaP and KCC2 in neonatal rat lumbar motoneurons
					• Acute inhibition of calpains restores the motoneuronal expression of Nav and KCC2, normalizes INaP and KCC2 function, and curtails spasticity
					• The calpain-mediated proteolysis of Nav and KCC2 leads to spasticity after SCI by driving the hyperexcitability of motoneurons
Li et al. (2022)	• Female adult Sprague–Dawley rats	• Incomplete SCI at T10	• TrkB-IgG	• 0.25 g/l	• Exercise training reduces the excitability of motoneurons and boosts the production of GAD-65, GAD-67, and KCC2 after SCI *via* TrkB signaling
			• BWSTT	• From the 8th day after SCI, 6 m/min for 20 min per session, twice a day, 5 days a week, for 4 weeks	
Allen et al. (2019)	• Adult male Lewis rats	• Unilateral cervical spinal cord contusion (C2SC)			• NKCC1 and KCC2 are present on phrenic motor neurons and may respond with transient dysregulation after spinal injury
Huang et al. (2017)	• Male Sprague-Dawley rats	• Spinal transections at T2	• BDNF	• i.t., 0.4 μg, 10 μl vol	• SCI changes how BDNF affects the expression of KCC2 and the function of GABA
			• capsaicin	• subcutaneous injections, 50 μl vol	• BDNF plays an essential role in blocking the development of spinally mediated nociceptive sensitization but does not reverse EMR
					• SCI leads to an increase in ERK/pERK expression while BDNF lowers the expression of ERK/pERK
Yan et al. (2018)	• Adult female (8-week old) Sprague-Dawley (SD) rats	• Acute SCI at T12	• TGN-020	• intra-peritoneally injection 200 mg/kg	• Decreasing the expression of AQP4 and NKCC1 can reduce spinal cord edema and neuronal loss after SCI
			• Bumetanide	• intra-peritoneally injection 3 mg/kg	
Lee et al. (2011)	• Adult male Sprague-Dawley rats	• Contusive SCI at T9			• During acute and chronic phases following SCI, the phosphorylation of NKCC1 and WNK1 undergoes a sustained increase
Mòdol et al. (2014)	• Adult male Sprague-Dawley rats	• Right sciatic nerve lesion	• Bumetanide	• Intraperitoneal injection daily, 30 mg/kg, from 1 to 16 dpi	• Changes in NKCC1 and KCC2 in DRG, spinal cord, and central pain areas contribute to the development of neuropathic pain
Mekhael et al. (2019)	• Adult female CD-1 mice	• SCI at T13	• TNF-α	• Lumbar intrathecal injection, 200 ng	• The upregulation of NKCC1 in mice leads to spasticity
			• MG-132	• Lumbar intrathecal injection, 375 ng	• a-tsDCS treatment can lead to a long-lasting reduction in spasticity following SCI *via* the downregulation of NKCC1
			• a-tsDCS	• 1.5 mA,20-min/day, 7 days	
López-Álvarez et al. (2015)	• Adult female Sprague-Dawley rats	• Right sciatic nerve lesion	• iTR session	• 1-h running, starting at 10 cm/s, increasing 2 cm/s every 5 min, until a maximal speed of 32 cm/s	• iTR normalizes the expression of NKCC1 and boosts that of KCC2
					• iTR reduces microgliosis in the L3–L5 dorsal horn
					• iTR increases BDNF expression in microglia 1–2 weeks post-injury
					• iTR prevents neuropathic pain by blocking collateral sprouting and NKCC1/KCC2 dysregulation

SCI, spinal cord injury; SNI, spated nerve injury; DIOA, (dihydroindenyl) oxy] alkanoic aci; BDNF, brain-derived neurotrophic factor; TrkB, tropomyosin-receptor-kinase-B; Cbl-b, Casitas B-lineage lymphoma b; PLCγ, Phospholipase C-γ; LSP, lipopolysaccharide; BWSTT, Body weight-supported treadmill training; a-tsDCS, anodal-sDCS; iTR, session,increasing-intensity treadmill exercise; i.t., intrathecal injection; i.p., intraperitoneal injection; LIFU, Low-intensity focused ultrasound.

### KCC2/NKCC1 and neuropathic pain after spinal cord injury

Neuropathic pain is a common complication after SCI and occurs in approximately 60%–80% of affected patients (Finnerup et al., 2001). Neuropathic pain has a severe social and psychological impact on patients with SCI, and is closely related to their poor overall health, poor quality of life, and high levels of depression (Anderson, 2004; Widerström-Noga, 2017). Pain after SCI is challenging to manage. Approximately 2/3 of the patients do not have a suitable treatment plan, especially for severe neuropathic pain. The latter is a strong predictor of a decline in quality of life after SCI, imposing a heavy burden on both families and society (Burke et al., 2017). Owing to the heterogeneity of etiology, differences in genetic susceptibility, and differences in environmental factors, it is difficult to predict which patients will have neuropathic pain and how patients will respond to specific therapeutic drugs (Kahle et al., 2014). The incomplete understanding of the molecular mechanism underlying neuropathic pain hinders the development of targeted interventions, which underscores the need to identify and develop novel therapeutic strategies for the treatment of this condition, particularly at the molecular level (Shiao and Lee-Kubli, 2018). The KCC2-/NKCC1- mediated regulation of chloride homeostasis has potential as a molecular target for the treatment of neuropathic pain after SCI (Cramer et al., 2008).

Changes in the levels of KCC2 and NKCC1, key factors in the maintenance of Cl^−^ homeostasis, have been implicated in the process of neuropathic pain after SCI (as shown in Figure 1) (Hasbargen et al., 2010; Fakhri et al., 2021). The expression of KCC2 was reported to be downregulated at the site of injury, accompanied by a transient and significant upregulation of NKCC1 expression, and this altered expression trend was consistent with the occurrence of post-neuropathic pain (Cramer et al., 2008). In addition, studies have found that the shift in NKCC1 and KCC2 expression in the spinal cord is also an important intermediate link in the development of neuropathic pain resulting from peripheral nerve injury (Coull et al., 2003; Mòdol et al., 2014; López-Álvarez et al., 2015).

GABA_A_Rs are involved in the regulation of the tonic-inhibitory effect in the dorsal horn of the spinal cord, maintaining the relative balance of inhibitory and excitatory systems in the central nervous network (Sivilotti and Woolf, 1994). After SCI, the function of GABA_A_Rs also changes to some extent, and their activation can produce depolarizing (excitatory) effects as well as promote the emergence of nociceptive sensitization (Huang and Grau, 2018). Pain sensitivity is reduced in rats with SCI when they are pretreated with GABA_A_R antagonists before the induction of SCI (Cramer et al., 2008). Similar treatments increase the sensitivity to nociceptive responses in healthy rats (Sorkin et al., 1998). Applying the GABA_A_R agonist muscimol to the spinal cord prevents thermal hyperalgesia after peripheral nerve injury (Miletic et al., 2003). Transplantation of GABAergic neuron precursors into the dorsal horn also reduces neuropathic pain (Bráz et al., 2012). However, existing GABA_A_R modulators, such as benzodiazepines or GABA_A_R agonists, are rarely used to treat neuropathic pain because of their narrow therapeutic window and associated adverse effects, such as sedation and hearing impairment (Kahle et al., 2014). Therefore, correcting the abnormal Cl^−^ concentration gradient in the dorsal horn of the spinal cord by targeting KCC2 and NKCC1 represents a promising therapeutic direction for restoring the inhibitory function of the GABAergic system and ultimately relieving or improving neuropathic pain.

The role of BDNF in neuropathic pain after SCI has also been intensively studied given that it functions upstream of KCC2. Inflammation or nerve injury can inhibit the expression and function of KCC2 in the dorsal horn of the spinal cord through the BDNF/TrkB signaling pathway and promote the occurrence and development of neuropathic pain (Rivera et al., 2002; Miletic and Miletic, 2008). Promoting BDNF expression in normal adult rats can downregulate the level of membrane-bound KCC2 and reduce the inhibitory effect of the GABAergic system (Huang et al., 2017). Meanwhile, promoting BDNF expression in rats with transected spinal cords can enhance the originally attenuated GABAergic inhibitory effect, which, in turn, can increase the expression of KCC2 and relieve hyperalgesia, which may associate with BDNF-/TRKB-induced upregulation of KCC2 expression (Wenner, 2014; Huang et al., 2017; Sanchez-Brualla et al., 2018). In a rat model of neuropathic pain, the administration of neuroheal was found to alter the sensory signal input of the dorsal root ganglia through the P2X4-BDNF-KCC2 pathway, thereby reducing neuropathic pain (Romeo-Guitart and Casas, 2020). Moreover, it has been reported that the effect of BDNF on pain may be mediated *via* the regulation of the ubiquitination level of KCC2 in the posterior horn of the spinal cord (Ma et al., 2021).

In addition to impaired motor and sensory pathways, 5-HT axons originating from the brainstem are often also impaired in function and display reduced receptor expression after SCI, leading to neural circuit reorganization (Khalki et al., 2018). Damaged 5-HT neurons also play a part in the process of chronic pain, spasms, and autonomic hyperreflexia after SCI (Fauss et al., 2022). The 5HT_2A/2B/2C_-specific agonist DOI can restore chloride homeostasis through the Ca^2+^-dependent PKC pathway (Bos et al., 2013). Meanwhile, specific activators of the 5-HT_2A_ receptor can exert an analgesic effect by enhancing the expression of KCC2 in spinal dorsal peduncle neurons (Sánchez-Brualla et al., 2018).

Transient receptor potential vanilloid 1 (TRPV1) is an important protein in the perception of pain (Takayama et al., 2015) and its expression in the dorsal horn of the spinal cord is an essential link in the emergence of neuropathic pain after SCI (Wu et al., 2013; Lee et al., 2021). The regulation of NKCC1 expression mediated by TRPV1 through the activation of the PKC/phosphorylated extracellular signal-regulated kinase (pERK) signaling pathway may be the mechanism underlying its involvement in SCI-related neuropathic pain (Deng et al., 2021).

### KCC2/NKCC1 and spasticity after spinal cord injury

The plasticity of nerves after SCI not only engenders beneficial changes in structure and function in the spinal cord but also leads to the appearance of spasticity, which occurs in approximately 60%–80% of affected patients (Finnerup, 2017). Spasticity after SCI can also lead to chronic pain and deformity of the musculoskeletal system, which subsequently affect the mood, sleep, quality of life, cognitive function, and recreational activities of patients, and can also greatly affect their rehabilitation (Andresen et al., 2016; Holtz et al., 2017). Common treatments for spasticity include drugs such as baclofen, chemical neurolysis, botulinum toxin injection, surgery, and electrical stimulation, all of which have drawbacks and differences in efficacy (Walter et al., 2002; Elbasiouny et al., 2010). Spasticity after SCI may share some afferent pathways with neuropathic pain; however, owing to differences in symptoms, studies on neuropathic pain focus more on sensory neurons in the superficial dorsal horn of the spinal cord, while investigations relating to spasticity focus more on motor neurons in the anterior horn. Therefore, its chloride homeostasis is mainly regulated by KCC2 (Côté, 2020).

Disruption of chloride homeostasis after SCI, especially the downregulation of KCC2 in motor neuron membranes, depolarizes the Cl^−^ equilibrium potential and reduces the strength of postsynaptic inhibition (Boulenguez et al., 2010). In healthy rats, pharmacological blockade of KCC2 can reduce the rate-dependent inhibition of the Hoffman reflex (rate-dependent depression, RDD) and produce spasticity like that seen in rats with SCI. Improvements in spasticity-like symptoms have also been reported (Boulenguez et al., 2010). When a KCC2 activator was administered to rats with spinal cord lesions, it was found that the expression of KCC2 was up-regulated in the membranes of motor neurons of the lumbar enlargement, and the spasticity symptoms were alleviated (Bilchak et al., 2021). Body Weight-Supported Treadmill training can also up-regulate KCC2 through the trkB pathway, regulating spinal cord excitability and reducing spasticity (Li et al., 2022). Additionally, (Mekhael et al., 2019), reported that transspinal direct current stimulation in injured mice can reduce spasticity and promote motor function recovery, and that this effect may be related to the downregulation of NKCC1, but not the upregulation of KCC2.

An increase in BDNF expression through exercise followed by the remodeling of KCC2 function was found to be an effective means of improving spasticity symptoms after SCI (Tashiro et al., 2015; Côté et al., 2014; Beverungen et al., 2020). The overexpression of the neurotrophic factors Neurotrofin-3 (NT-3), and Insulin-like growth factor 1 (IGF-1) has also been suggested as a treatment for spasticity after SCI, and their efficacy was speculated to be related to the functional remodeling of KCC2 (Chang et al., 2019; Talifu et al., 2022). In contrast to that found in the neuropathic pain model after SCI, Ryu et al. reported that an increase in the levels of 5-HT_2A_ after SCI did not significantly affect spasticity symptoms or KCC2 expression in motor neurons (Ryu et al., 2021).

When TCB-2 is used to specifically activate the 5-HT_2A_ receptor, it can also be stabilized by KCC2-mediated chloride ion, relieving spasticity after SCI (Bos et al., 2013). Although the downregulation of KCC2 begins within 24 h after SCI, spasticity only appear weeks or even months later, possibly because of the presence of persistent inward currents (PICs), PICs require prolonged depolarization to be fully activated and are inhibited by hyperpolarization (Li et al., 2004; Bras and Liabeuf, 2021). The depolarization of excitatory postsynaptic potentials (EPSPs) following KCC2 downregulation may allow sensory input-evoked depolarization to activate PICs and these may fail to inactivate owing to the inability to hyperpolarize, which ultimately induces spasticity (Li et al., 2004; Bras and Liabeuf, 2021). In a neonatal rat model of SCI, it was also found that calpain can up-regulate the sustained sodium ion current in motor neurons and downregulate KCC2 expression after SCI, resulting in the appearance of spasticity (Plantier et al., 2019). Combined, these observations suggest that the downregulation of KCC2 and the upregulation of PICs may play a synergistic role in the pathophysiology of spasticity.

In brief, the downregulation of KCC2 expression below the injury site after SCI, especially in motor neuron membranes, can depolarize the intracellular membrane potential and exert excitatory effects, and may represent an important component in the occurrence of spasticity (Boulenguez et al., 2010; Bilchak et al., 2021). BDNF, exercise training, or regulation of neurotrophic factor levels can improve spasticity by promoting KCC2 expression (Tashiro et al., 2015; Ma et al., 2021; Talifu et al., 2022).

### KCC2 and motor function after spinal cord injury

Substantial evidence supports the use of exercise training in current rehabilitation regimes for the functional recovery of spinal cord injuries (Harvey, 2016). The restoration of chloride homeostasis mediated by NKCC1 and KCC2 may be an important physiological basis for the associated improvement in spinal cord functions (Côté et al., 2014).

The regulation of KCC2 has a unique role in the recovery of motor function after SCI, making this co-transporters an important target for motor reconstruction in the spinal cord following injury (Chen et al., 2018). The administration of the KCC2-specific activator CLP290 could significantly restore the coordinated step function of mice that had completely lost descending control; similar functional recovery was also achieved by adeno-associated virus-mediated overexpression of KCC2 (Chen et al., 2018). However, the authors also indicated that KCC2 promotes the recovery of motor function, but in a manner different from that involved in the relief of spasticity and neuropathic pain, that is, promoting the expression of KCC2 in inhibitory interneurons is the key to the reconstruction of motor function (Chen et al., 2018). Increased KCC2 expression can also be achieved by overexpressing BDNF; however, this approach may have complications, such as spasticity, resulting from motor neuron hyperexcitation (Ziemlińska et al., 2014). The positive and quantitative regulation of KCC2 to promote the recovery of motor function may become the focus of research for SCI rehabilitation.

At present, there is no convincing evidence regarding the existence of a relationship between NKCC1 expression and motor function after SCI. In animal models of brain injury, the long-term administration of the NKCC1-specific inhibitor bumetanide in rats can enhance axonal bud growth after focal cerebral ischemia. In addition, bumetanide treatment can up-regulate KCC2 and BDNF levels, downregulate those of NKCC1, and improve exercise-related behavior in rats after stroke (Mu et al., 2017). Another report also showed that bumetanide improves sensory and motor recovery after traumatic brain injury (Zhang et al., 2017). The results of these studies suggested that in animal models of traumatic SCI, the downregulation of NKCC1 levels may also have the potential to improve neurological outcomes, although this effect may be achieved indirectly through the modulation of chloride homeostasis. Endogenous serotonin levels may also affect the alternate movement pattern of lower limbs by affecting chloride ion homeostasis and the GABAergic system, which is also a potential target for motor reconstruction after SCI (Tang, 2020).

## Conclusion

Numerous basic studies have confirmed the notable therapeutic potential of NKCC1 and KCC2 in neuropathic pain, spasticity, and motor function recovery after SCI, and these co-transporters are expected to become key targets in future SCI treatment (Boulenguez et al., 2010; Chen et al., 2018).

To achieve this, key pathways are blocked/activated by pharmacological means, such as the P2X4-BDNF-KCC2 pathway (Ferrini et al., 2013; Romeo-Guitart and Casas, 2020), BDNF-trkb (Miletic and Miletic, 2008; Rivera et al., 2002; Rivera et al., 2004; Huang et al., 2017; Wenner, 2014; Sánchez-Brualla et al., 2018) or direct regulation of KCC2 activity, such as N and C termin (Ferrini et al., 2013) N-terminal loop Chi et al., 2021, phosphorylation of serine 940 (Leonzino et al., 2016) will likely be potential targets for clinical treatment of spinal cord injury and its complications. In addition, through exercise training, modulation of the 5-HTergic system (Bos et al., 2013; Sánchez-Brualla et al., 2018; Ryu et al., 2021), the GABAergic system, and TRPV1 (Deng et al., 2021) to indirectly affect NKCC1 and KCC2 expression levels are also feasible intervention strategies. The targeted regulation of different types of neurons may become a future treatment strategy for SCI and its complications (Chen et al., 2018). As KCC2 and NKCC1 are widely distributed in the nervous system, how to achieve localization, orientation, and quantitative regulation of their levels may be the biggest obstacle to their clinical application in the treatment of SCI. It is necessary to solve these problems through more in-depth and comprehensive research for the benefit of patients with SCI.
